# Deformation rate of engineered wood flooring with response surface methodology and adaptive network-based fuzzy inference system

**DOI:** 10.1371/journal.pone.0292815

**Published:** 2023-10-12

**Authors:** Huixiang Wang

**Affiliations:** Department of Biological Sciences, XinZhou Normal University, Xinzhou, Shanxi, PR China; University of Ljubljana Biotechnical faculty: Univerza v Ljubljani Biotehniska fakulteta, SLOVENIA

## Abstract

Controlling the deformation rate is the key to improving the product quality of engineered wood flooring. In this work, the changes in the deformation rate of engineered wood flooring were in focus with cold-pressing, response surface methodology, and adaptive network-based fuzzy inference system were used to explore the relationship between deformation rate and processing parameters, including adhesive spreading rate, pressing time, and pressing pressure. According to the results, the deformation rate was positively related to pressing time, while it increased first and then decreased with both the increase of adhesive spreading rate and pressing pressure. Meanwhile, a mathematical model was developed, and the significant influence of each term on the deformation rate was analyzed. This model had high feasibility and can be used to describe the relationship between the deformation rate and processing parameters. Furthermore, an adaptive network-based fuzzy inference system model was established. It has higher accuracy than that of the response surface methodology model, and it can be used for predicting deformation rate and optimizing processing parameters. Finally, an optimal processing conditions with the lowest deformation rate was determined as follows: 147 g/m^2^ adhesive spreading rate, 12s pressing time, and 1.2 MPa pressing pressure, and it hope to be adopted in the industrial processing of engineered wood flooring with respective of the higher product quality and lower production costs.

## 1. Introduction

Engineered wood flooring is a new wooden product originating in Northern Europe. With the rapid development of preparation technology, engineered wood flooring is popular globally because of its stable quality, beautiful texture, and easy installation and maintenance [1, 2]. Engineered wood flooring is made by interlacing and laminating boards with gluing and pressing processes, including the top-surface and under-core boards. The top-surface board, also called the wear layer, mainly uses hardwood, such as Oak, Walnut, etc. The under-core board is mainly made of plywood or solid wood panels, mainly made of Poplar, Pine, and so on [3–5].

In the industrial manufacturing of engineered wood flooring, the warping deformation is an important indicator for judging its quality of dimensional stability [2, 6, 7]. Warping is the bending deformation of the initial board, and it has always been a research hotspot in wood composite materials [8]. A finite element model was established by Blanchet et al. [9]. Based on the validation test, their work showed that the developed model has high accuracy and can be used for the product design of engineered wood flooring. Meanwhile, the changes in warping deformation of engineered wood flooring were explored by Chen et al. [10] at different processing variables of wood shapes, wood structures, and decorative veneer types, and an optimal flooring structure was determined in terms of the lower warping deformation. In the related work, Guo et al. [11] also investigated the influence of wood shapes, structures, and decorative veneer types on dimensional stability. Their work indicates that the engineered wood flooring decorated by birch with mono-block veneer has the greatest dimensional stability. Furthermore, the dimensional stability, modulus of elasticity, and bonding strength of engineered wood flooring were studied by Zhou et al. [12]. They found that the adhesive spreading rate, pressing time, temperature and pressure had great impact on the dimensional stability, modulus of elasticity and bonding strength, and controlling the values of adhesive spreading rate, pressing time, temperature and pressure is the key to improve the material properties of engineered wood flooring.

Response surface methodology (RSM) is a collection of mathematical and statistical techniques, and it is widely adopted for modeling and analyzing [13]. RSM was used by Wang et al. [14] to explore the effect of processing parameters on the shear strength of engineered wood flooring. In their work, a predicted model was developed to find the relationship between shear strength and pressing time, pressure, and adhesive spreading rate. Meanwhile, the material properties of rubber-wood particles reinforced cement-based composites were studied by Zhao et al. [15] using RSM, and the optimal processing variables were determined with the multi-objective of internal bond strength, modulus of elasticity, and flexural strength. In addition to material properties research, RSM was applied in other fields [16, 17], such as mechanical processing, biochemistry, etc. Thus, RSM is a great research method that can be used for developing mathematical models, predicting experimental results, and optimizing parameters [18].

Adaptive Network-based Fuzzy Inference System (ANFIS) was first proposed by Jang [19] based on the Takagi Sugeno model. ANFIS implements three basic processes using neural networks: fuzzification, fuzzy inference, and anti-fuzzification of fuzzy control [20]. The learning mechanism of neural networks is used to automatically extract rules from input and output sample data, forming an adaptive neural fuzzy controller [21]. The fuzzy inference control rules are self-adjusted by offline training and online learning algorithms, making the system develop in the direction of adaptation, self-organization, and self-learning. ANFIS also has been widely used in the research of material properties [22, 23].

Controlling the deformation of the engineered wood flooring is the key to improving product quality. The related reports show that RSM [24] and ANIFIS [25] are great methods. They can potentially be used to explore the changes in the deformation of engineered wood flooring at different processing conditions. Currently, research on the deformation of engineered wood flooring is limited. In the production process, how to suppress floor deformation has always been an urgent problem that manufacturing enterprises need to solve.

To this end, this work aims to improve the quality of engineered wood flooring. RSM and ANFIS were used to explore the changes in deformation rate at different processing conditions, and the optimal processing parameters were determined. This work is hoped to provide scientific support for the industrial processing of engineered wood flooring.

## 2. Materials and methods

### 2.1 Materials

**Fig 1:**
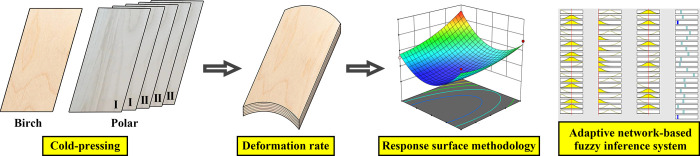
Experimental diagram.

As shown in Fig 1 and Table 1, Birch (*Betula spp*.) was used as the surface board, and the substrate material was made of Poplar (*Populus sp*.) with different thicknesses. Meanwhile, polymer isocyanate adhesive (API) was prepared by (Foshan Gubiliao Chemical Technology Co., Ltd, Guangdong, China), it was used for the preparation of engineered wood flooring.

**Table 1:** Material properties and dimensions of veneer materials.

Veneer materials	Density	Moisture content	Thickness	Length	Width
Birch	624 kg/m^3^	8.0%	3.6 mm	900 mm	160 mm
Poplar I	366 kg/m^3^	8.1%	3.6 mm	900 mm	160 mm
Poplar II	366 kg/m^3^	8.1%	3.6 mm	900 mm	160 mm

### 2.2 Experimental design

RSM and ANFIS were used to explore the changes in deformation rate at different conditions (Fig 1). Table 2 displays the experiment design. The processing variables of adhesive spreading rate, pressing pressure, and time were in focus, and their levels were determined based on the related work and industrial processing of engineered wood flooring.

**Table 2:** Experimental design.

Runs	Adhesive spreading rate *R* (g/m^2^)	Pressing time *T* (s)	Pressing pressure *P* (MPa)	Deformation rate *f*_*w*_ (%)
1	140	12	1	0.1831
2	140	12	1.2	0.1137
3	140	12	1.4	0.1863
4	140	16	1	0.1459
5	140	16	1.2	0.1173
6	140	16	1.4	0.2188
7	140	20	1	0.1831
8	140	20	1.2	0.1569
9	140	20	1.4	0.2663
10	155	12	1	0.1576
11	155	12	1.2	0.8641
12	155	12	1.4	0.1290
13	155	16	1	0.1494
14	155	16	1.2	0.9110
15	155	16	1.4	0.1710
16	155	20	1	0.1818
17	155	20	1.2	0.1382
18	155	20	1.4	0.2242
19	170	12	1	0.2124
20	170	12	1.2	0.1231
21	170	12	1.4	0.1577
22	170	16	1	0.1996
23	170	16	1.2	0.1411
24	170	16	1.4	0.2054
25	170	20	1	0.2418
26	170	20	1.2	0.1789
27	170	20	1.4	0.2739

**Fig 2:**
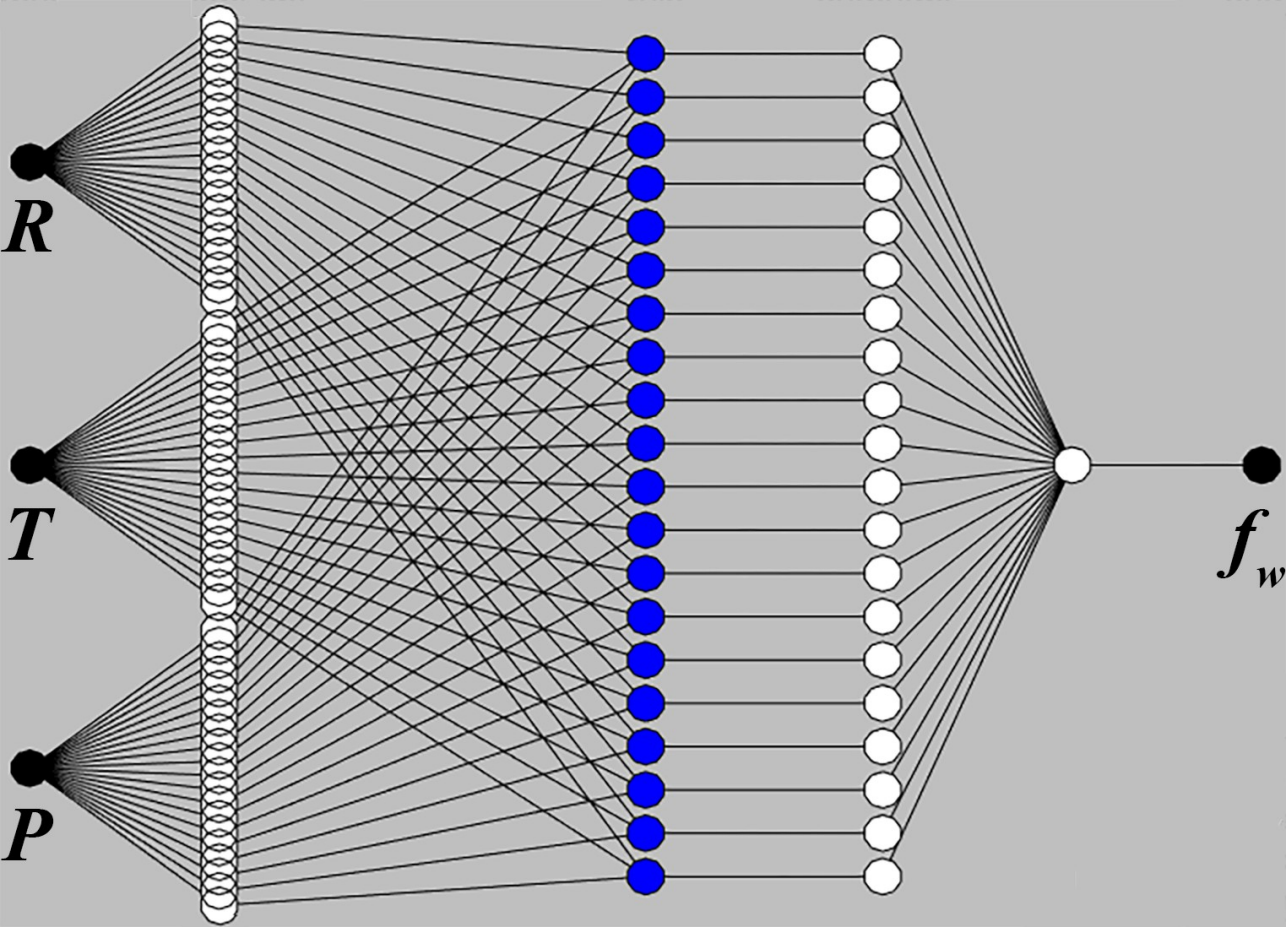
ANIFIS model structure.

Fig 2 shows the ANIFIS model structure. Twenty groups of experimental results (74%) were used as the training data, and seven experimental results (26%) were used as testing data. The input variables were adhesive spreading rate, pressing time, and pressing pressure, and the output result was the deformation rate of engineered wood flooring.

In this work, Sugeno-ANFIS [26]was adopted according to the If-Then rule:

$$If\;x_{1}\;is\;A_{1},\;and\;if\;x_{2}\;is\;B_{1},\;\mathrm{then}\;f_{1}=p_{1}x_{1}+q_{1}x_{2}+r_{1}$$

$$If\;x_{1}\;is\;A_{2},\;\mathrm{and}\;if\;x_{2}\;is\;B_{2},\;\mathrm{then}\;f_{2}=p_{2}x_{1}+q_{2}x_{2}+r_{2}$$

$$If\;x_{1}\;is\;A_{3},\;\mathrm{and}\;if\;x_{2}\;is\;B_{3},\;\mathrm{then}\;f_{3}=p_{3}x_{1}+q_{3}x_{2}+r_{3}$$

**Fig 3:**
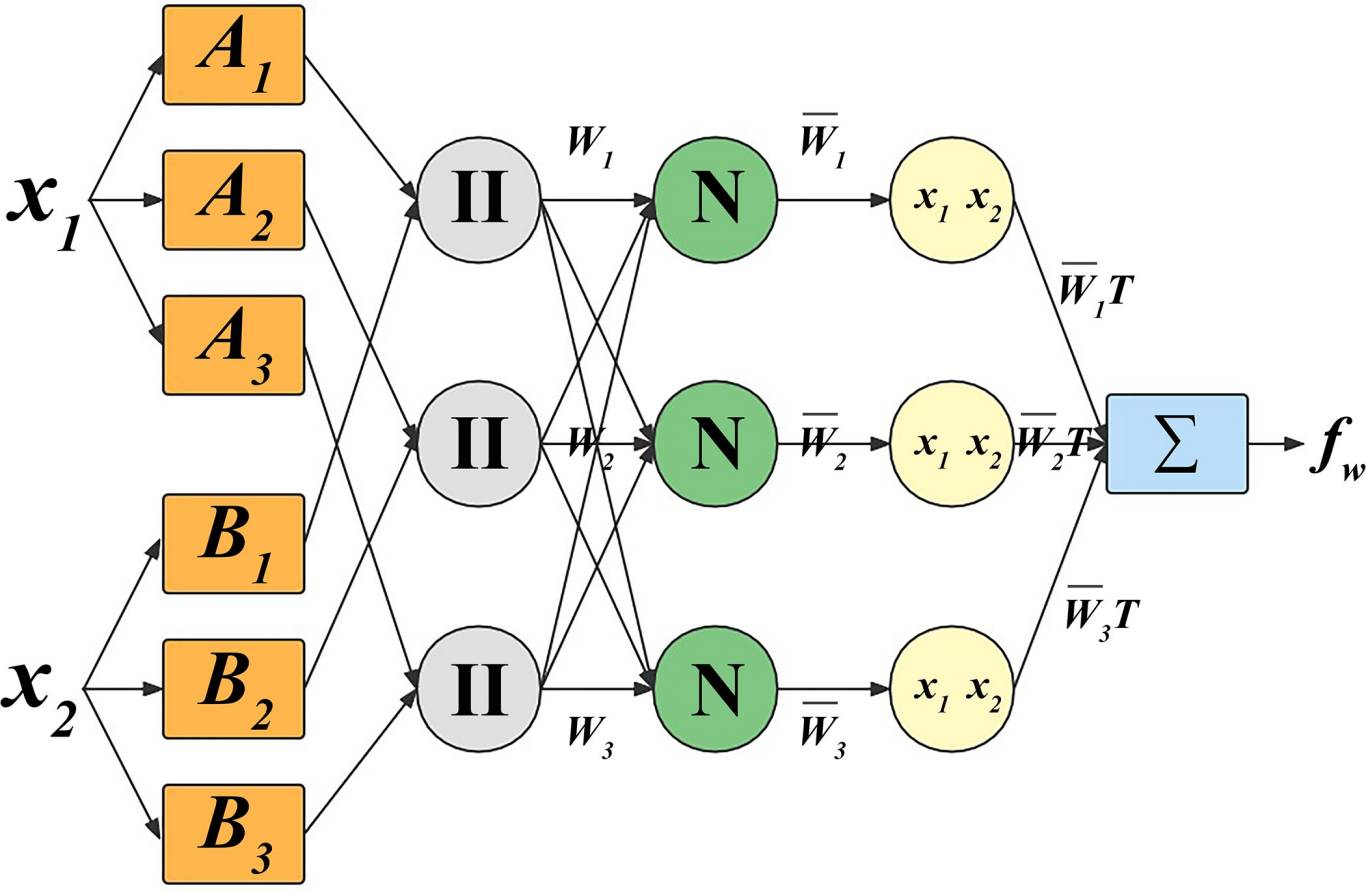
Schematic diagram of Sugeno-ANFIS.

Fig 3 displays the Sugeno-ANFIS structure with six layers, including input, fuzzification, product, normalization, defuzzification, and output layers [27]. In the input layer, adhesive spreading rate, pressing time, and pressing pressure were selected as the input variables and fed into the ANFIS model. This model utilizes recursive backpropagation and forward propagation over several epochs to adjust its weights, continuously reducing loss and increasing accuracy. A lower epoch count indicates faster convergence. The model identifies the most and least essential features based on their correlation with the output or target label in the variables.

In the fuzzification layer, the input variable is converted into the membership of each fuzzy set, and each node is represented by a node function of Eq 1 [20].

$$\begin{array}{l} O_{1,i}=\mu A_{i}(x_{1})\\ O_{1,i}=\mu B_{i}(x_{2})\\ O_{1,i}=\mu C_{i}(x_{3}) \end{array}$$ (Eq 1)

where *O*_*1*,*i*_ is the deformation rate, and *μA*_*i*_(*x*_*1*_), *μB*_*i-2*_(*x*_*2*_), and *μC*_*i-4*_(*x*_*3*_) are Gaussian membership functions.

The product layer shows the rule applicability, where the output of each node is the product of the input signals (Eq 2) [20]. Meanwhile, a weight function of Eq 3 was used to describe the normalized rule firing strengths.


$$O_{2,i}=w_{{}^{{}_{i}}}=\mu A_{i}(x_{1})B_{i}(x_{2})C_{i}(x_{3})$$ (Eq 2)

$$O_{3,i}=\bar{w_{{}^{{}_{i}}}}=\frac{w_{i}}{w_{1}+w_{2}+w_{3}}$$ (Eq 3)

In the layer of defuzzification, the crisp values can be obtained from the fuzzy values with the adaptive nodes by Eq 4 [20].


$$O_{4,i}=\bar{w_{{}^{{}_{i}}}}(p_{{}^{{}_{i}}}x_{1}+q_{{}^{{}_{i}}}x_{2}+r_{{}^{{}_{i}}})$$ (Eq 4)

In the layer of the output layer, the deformation rate was used as the output variable and was acquired by Eq 5 [20].


$$O_{5,i}=\sum_{i}\bar{w_{{}^{{}_{i}}}}T_{i}=\frac{\sum w_{{}^{{}_{i}}}T_{i}}{\sum w_{{}^{{}_{i}}}}$$ (Eq 5)

### 2.3 Experimental measurement

Measurement standards for the deformation rate of engineered wood flooring were performed according to the GB/T 18103–2013 (Test methods of evaluating the properties of wood-based panels and surface decorated wood-based panels). The deformation rate can be obtained based on Eq 6.

$$f_{w}=\frac{H_{max}}{W}\times 100\%$$ (Eq 6)

where *f*_*w*_ is the deformation rate, *H*_*max*_ stands for the maximum chord height in mm, and *W* donates the width in mm of engineered wood flooring. Each processing combination was repeated five times, and the average values were used to describe the deformation rate of engineered wood flooring.

## 3. Results and discussion

### 3.1 Analysis of deformation rate with response surface methodology

Changes in the deformation rate of engineered wood flooring were analyzed by using RSM, and a quadratic model was developed, as shown in Eq 7.

$$\begin{array}{l} f_{w}=+5.77-0.04R-0.06T-3.37P+5.25\times 10^{-5}RT-5.59\times 10^{-3}RP\\ \;+0.02TP+1.58\times 10^{-4}R^{2}+1.02\times 10^{-3}T^{2}+1.64P^{2} \end{array}$$ (Eq 7)

where *f*_*w*_ is the deformation rate in %, *R* stands for the adhesive spreading rate in g/m^2^, and *T* donates the pressing time in s, and *P* is the pressing pressure in MPa.

In order to verify the feasibility of the developed model, standard deviation, coefficient of variation, *R*^*2*^ and Adjusted-*R*^*2*^ were used to make fit statistics as given in Table 3 [28]. Standard deviation describes the degree of dispersion of a dataset, and coefficient of variation is the ratio of the original data’s standard deviation to the original data’s mean. They all had a low value. Meanwhile, the values of *R*^*2*^ and Adjusted-*R*^*2*^ were all close to 1. They all showed a good correlation between predicted and measured deformation rate values.

**Table 3:** Model fit statistics of deformation rate.

Standard deviation	Coefficient of variation	*R* ^*2*^	Adjusted-*R*^*2*^
9.17×10^−3^	6.26%	0.98	0.96

**Fig 4:**
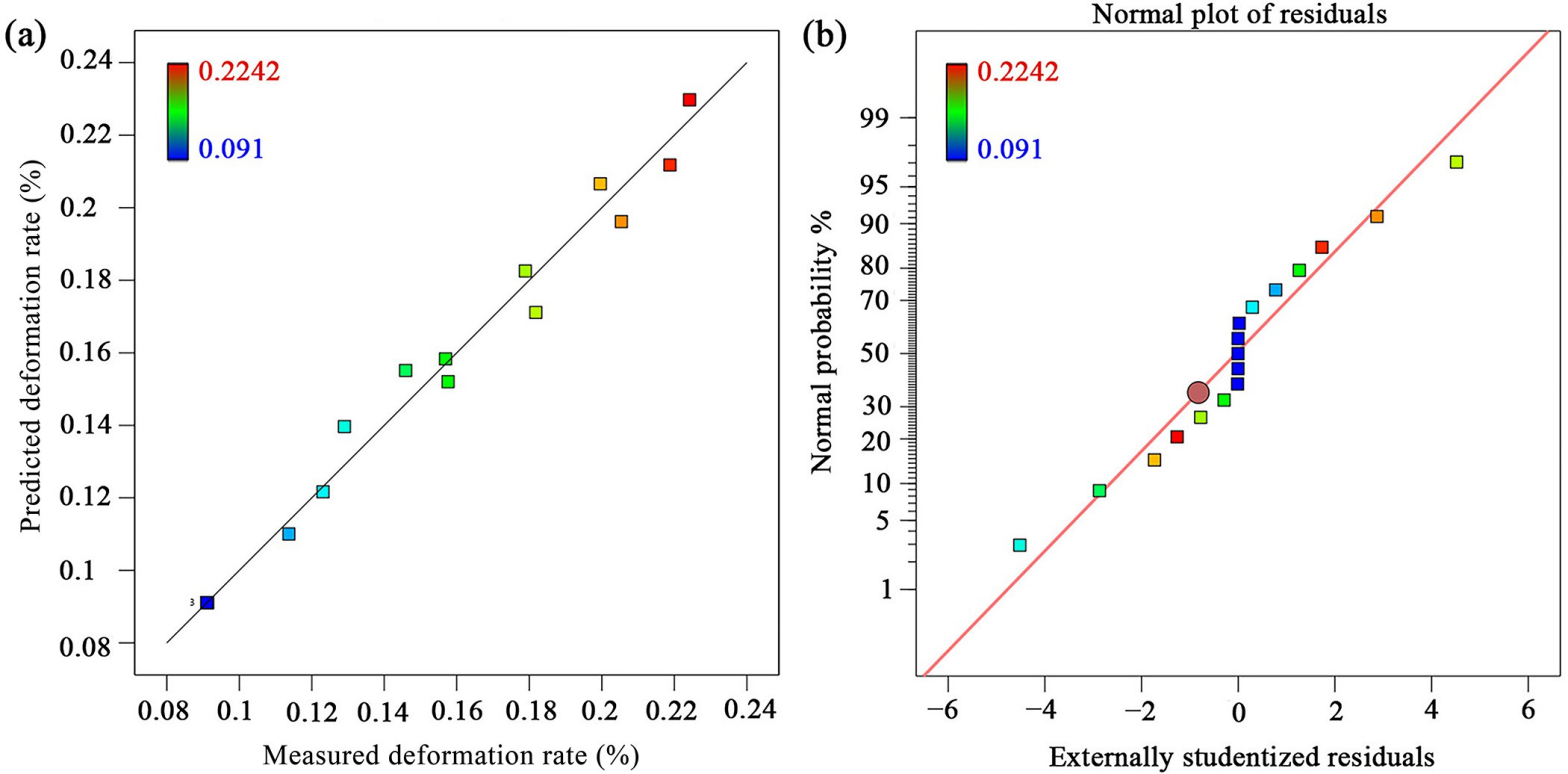
(a) Correlation between the measured and predicted values of deformation rate, (b) Normal distribution.

Furthermore, the correlation graph for the predicted and actual deformation rate is displayed in Fig 4A. It can be found that all the actual value (colored dots) is very close to the predicted line. In general, the developed model had high feasibility, and it can be used to describe the relationship between the deformation rate and processing parameters.

The primary assumption for conducting analysis of variance (ANOVA) [29] is that the data has the characteristics of normal distribution. Fig 4B shows that the deformation rate is concentrated in the setting range, revealing that the data has normality normal distribution characteristics. Therefore, ANOVA can be further used to analyze the developed model of Eq 7. The results of analysis of variance (ANOVA) with a significance level of 5 percent (α = 0.05) for the developed model was shown in Table 4, *i*.*e*., If the p-value of a term is lower than 0.05, this term can be considered as significant, otherwise insignificant [30]. Thus, the results suggest that the developed model of Eq 7 is statistically fit for the data: F-value = 48.58, p < 0.05. There is only a 0.01% chance that an F-value this large could occur due to noise.

**Table 4:** ANOVA of deformation rate.

Source	Sum of square	df	Mean Square	F-value	p-value	Remark
Model	0.037	9	4.08×10^−3^	48.58	< 0.0001	Significant
*R*	6.43×10^−4^	1	6.43×10^−4^	7.64	0.0279	Significant
*T*	5.96×10^−3^	1	5.96×10^−3^	70.92	< 0.0001	Significant
*P*	1.07×10^−3^	1	1.07×10^−3^	12.72	0.0091	Significant
*R*×*T*	3.97×10^−5^	1	3.97×10^−5^	0.47	0.5141	Insignificant
*R*×*P*	1.13×10^−3^	1	1.13×10^−3^	13.39	0.0081	Significant
*T*×*P*	1.26×10^−3^	1	1.26×10^−3^	14.99	0.0061	Significant
*R* ^*2*^	5.35×10^−3^	1	5.35×10^−3^	63.66	< 0.0001	Significant
*T* ^*2*^	1.13×10^−3^	1	1.13×10^−3^	13.43	0.0080	Significant
*P* ^*2*^	0.02	1	0.02	215.88	< 0.0001	Significant
Residual	5.88×10^−4^	7	8.41×10^−5^			
Cor total	0.04	16				

Furthermore, the values of variables of *R*, *T* and *P*, two-level interaction effects of *R*×*P* and *T*×*P*, and products of *R*^*2*^, *T*^*2*^ and *P*^*2*^, they were all less than 0.05, it can be obtained that those terms had a significant impact on the deformation rate. However, only the two-level interaction effects of *R*×*T* is higher than 0.05, and it has the insignificant contribution to the deformation rate.

**Fig 5:**
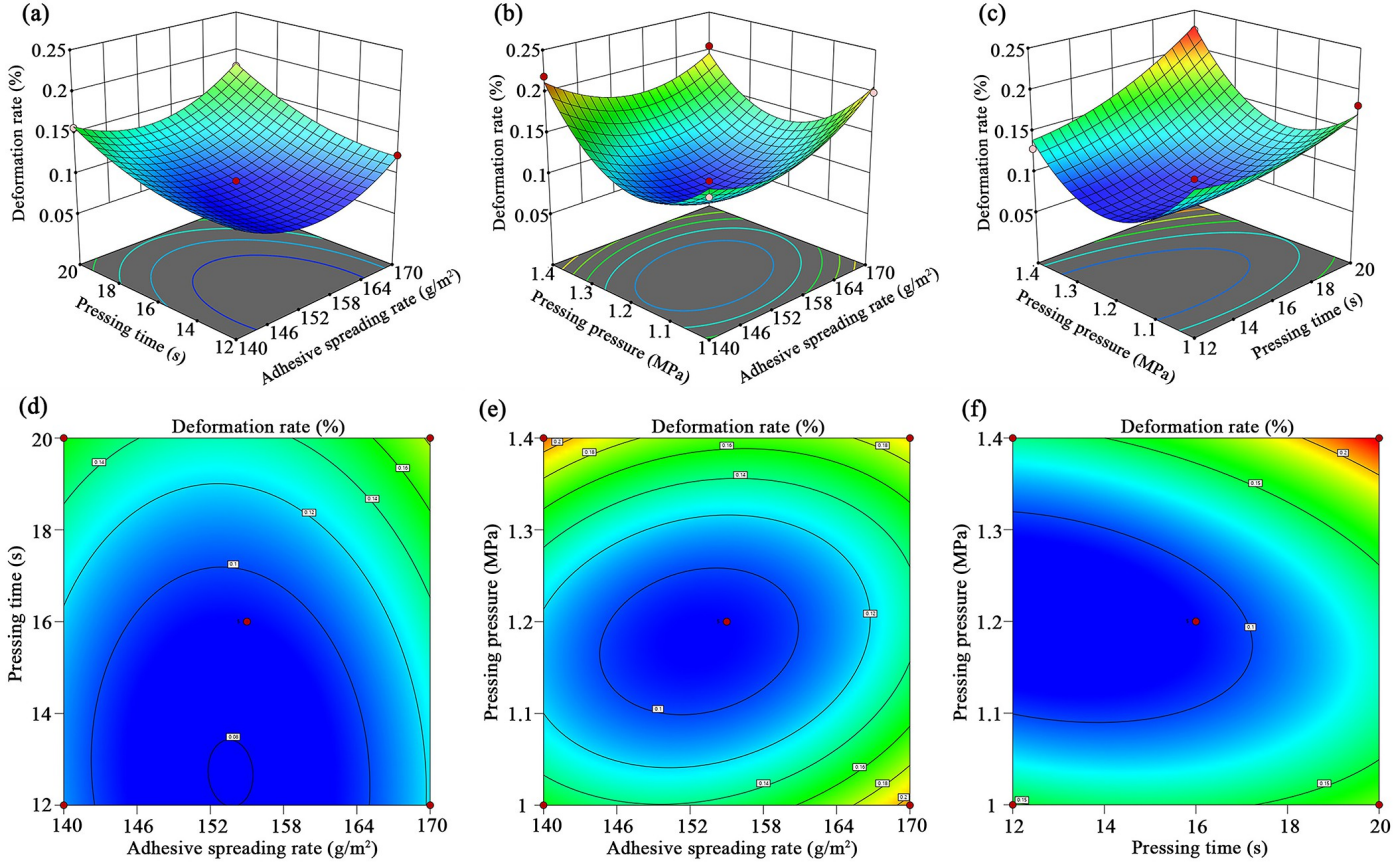
Three-dimension response surface (a-c) and contours (d-f) of two-level interactions on deformation rate.

Fig 5A–5C shows the effect of different processing parameters on the deformation rate. It can be found that the deformation rate increased first and then decreased with the increase of adhesive spreading rate. When the adhesive spread was low, there was not enough adhesive to form a continuous adhesive layer, and the bonding strength between the surface plate and substrate was low, affecting its dimensional stability. As the spread rate of the adhesive applied continued to increase, the deformation decreased. However, as the spread rate of adhesive applied continued to increase, the adhesive layer became too thick, which reduced the bonding performance of the boards, thereby reducing the dimensional stability of the floor and increasing the deformation.

Meanwhile, it can be obtained that the deformation rate was positively related to the pressing time. With the extension of cold pressing time, the adhesive had enough time to penetrate, forming a continuous and durable adhesive layer. The substrate and surface board were well bonded, and the dimensional stability increased. Finally, the changes in deformation rate also increased first and then decreased with the increase of pressing pressure. The increased pressing pressure made the adhesive layer fully in contact with the boards’ surfaces, resulting in a lower deformation rate. However, as the pressing pressure continued to increase, the higher pressing pressure may destroy the wood fiber with a counterproductive effect. Thus, the deformation rate increased first and then decreased as the pressing pressure increased.

Fig 5D–5F also indicates the two-level interaction effects of processing variables on the deformation rate. Based on the density of the contour map, the deformation rate was mainly affected by the pressing time in the interaction effects of adhesive spreading rate and pressing time. Meanwhile, pressing pressure mainly impacted the deformation rate in the interaction effects of adhesive spreading rate and pressing pressure. Finally, the deformation rate was mainly affected by the pressing time in the interaction effects of pressing time and pressure.

### 3.2 Analysis of deformation rate with adaptive network-based fuzzy inference system

**Fig 6:**
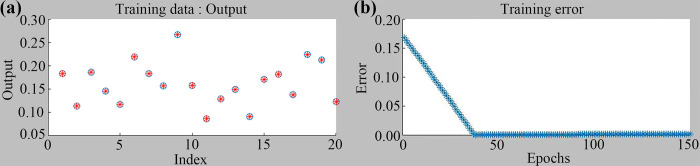
Training data and predicting error of deformation rate with ANFIS model.

According to the experimental results in Fig 6, A Fuzzy Inference System (FIS) with grid partition was implemented and trained for 150 learning epochs. The ANFIS model achieved saturation after 37 epochs, at which the error value remained constant. Specifically, the training error changes converged and reached an error value of 1.98×10^−3^, less than 0.001. Meanwhile, based on the predicted results with input-output pairs by the ANFIS model, it can be found that the predicted deformation rate values were close to the actual values, and its average error was equal to 1.50×10^−7^, which is lower than that of the RSM model. Thus, it can be obtained that the ANFIS model has higher accuracy than the RSM model, and it can be used to describe the relationship between the deformation rate and processing variables.

**Fig 7:**
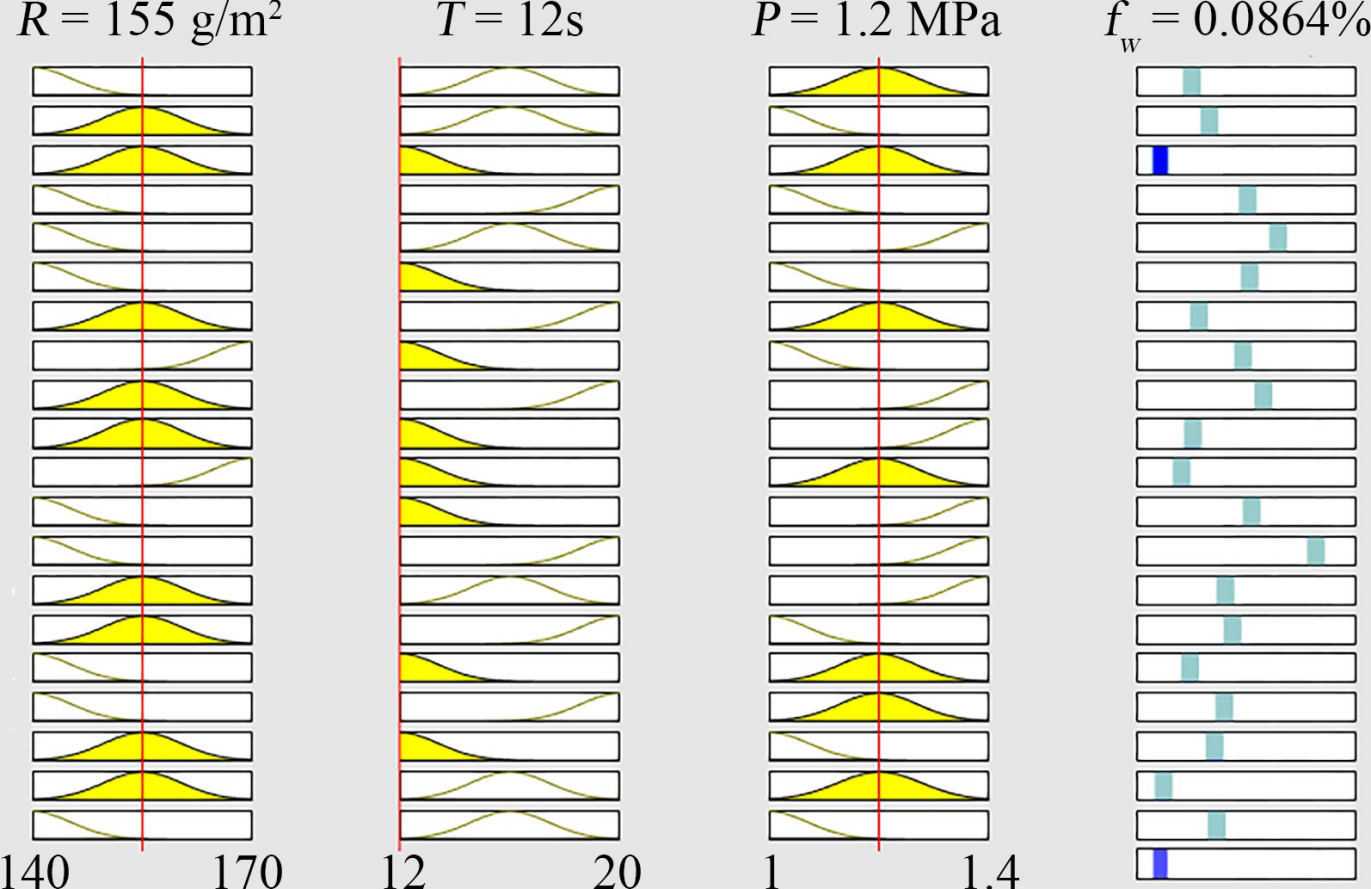
Sugeno inference system for deformation rate.

The Sugeno inference system (Fig 7) was used to define the correlation between the deformation rate and processing variables. As given in the Sugeno inference system with the ANFIS model, 27 rules have been adopted for the deformation rate. The first three columns are the processing parameters of adhesive spreading rate in g/m^2^, pressing time in s, and pressing pressure in MPa, and the last column is the output result of the deformation rate. Following this reasoning process, inferences can be made based on input parameters regarding the ANFIS’s predictive function. The height of the yellow area within the triangle stands for the membership values of fuzzy sets. Based on the ANFIS result, when the input processing variables of adhesive spreading rate, pressing time, and pressing pressure were equal to 155 g/m^2^, 12s, and 1.2 MPa, respectively, the third proportion of reasoning rule is lowest with the deformation rate of 0.09.

### 3.3 Optimization and verification of processing parameters

In the industrial processing of engineered wood flooring, the deformation rate is crucial for the evaluation index. It directly influences the final product quality [31, 32]. Thus, the lowest deformation rate for engineered wood flooring was in focus. As displayed in Table 5, based on the developed RSM model of Eq 7, the predicted lowest deformation rate of 0.901% can be obtained at the processing variables of 147 g/m^2^ adhesive spreading rate, 12s pressing time and 1.2 MPa pressing pressure, and at those variables, the actual average deformation was equal to 0.0843%. It can be calculated that its error rate was 6.88%. According to the ANFIS model, when at the processing variables of 155 g/m^2^ adhesive spreading rate, 12s pressing time, and 1.2 MPa pressing pressure, the predicted and actual lowest deformation rate can be obtained as 0.0864% and 0.0829%, respectively. The ANIFS model had a lower error rate (4.22%) than the RSM model (6.88%). This result is consistent with the previous result in the section 3.2.

**Table 5:** Optimization and verification results for RSM and ANFIS models.

Models	Adhesive spreading rate	Pressing time	Pressing pressure	Predicted deformation rate	Actual deformation rate	Error rate
RSM	147 g/m^2^	12s	1.2 MPa	0.0901%	0.0843%	6.88%
ANFIS	155 g/m^2^	12s	1.2 MPa	0.0864%	0.0829%	4.22%

According to the measurement standard of engineered wood flooring (Test methods of evaluating the properties of wood-based panels and surface decorated wood-based panels, GB/T 18103–2013), the deformation rate in the width direction must be less than or equal to 0.02%, and both the deformation rates results optimized by RSM and ANFIS models meet the standards of engineered wood flooring. Considering production costs, the optimal processing conditions were determined: adhesive spreading rate of 147 g/m^2^, pressing time of 12s, and pressing pressure of 1.2 MPa. Meanwhile, the combination of parameters was proposed to be applied in the industrial processing of engineered wood flooring with lower deformation rate and production costs.

## 4. Conclusions

In this work, a cold-pressing experiment was carried out to explore the deformation rate of engineered wood flooring, and RSM and ANFIS were used to investigate the effect of adhesive spreading rate, pressing time, and pressure on the deformation rate. The main conclusions are listed as follows:

1. The deformation rate increased first and then decreased with both the increase of adhesive spreading rate and pressing pressure, and it showed an increasing trend with the increase of pressing time.
2. A mathematical model was developed using RSM, and it has high feasibility and can be used to describe the relationship between the deformation rate and processing parameters. In this model, the terms of *R*, *T*, and *P*, the two-level interaction effects of *R*×*P* and *T*×*P*, and the products of *R*^*2*^, *T*^*2*,^ and *P* significantly impacted the deformation rate.
3. An ANFIS model was obtained, which has higher accuracy than the RSM model. ANFIS model can also be adopted for the prediction of deformation rate and the optimization of processing parameters.
4. Comprehensive comparison of the RSM and ANIFS model results, the optimal processing conditions were determined with the lowest deformation rate, where the adhesive spreading rate was 147 g/m^2^, pressing time was 12s, and pressing pressure was 1.2 MPa. This combination of processing parameters was proposed to be used in manufacturing engineered wood flooring for higher product quality and lower production costs.
